# A comparison of three treatment strategies in recent onset non-systemic Juvenile Idiopathic Arthritis: initial 3-months results of the BeSt for Kids-study

**DOI:** 10.1186/s12969-017-0138-4

**Published:** 2017-02-06

**Authors:** P. C. E. Hissink Muller, D. M. C. Brinkman, D. Schonenberg, Y. Koopman-Keemink, I. C. J. Brederije, W. P. Bekkering, T. W. Kuijpers, M. A. J. van Rossum, L. W. A. van Suijlekom-Smit, J. M. van den Berg, C. F. Allaart, R. ten Cate

**Affiliations:** 1 Department of Pediatrics/Pediatric Rheumatology, Leiden University Medical Center, Leiden, The Netherlands; 2 Department of Pediatrics, Alrijne Hospital Leiderdorp, Leiderdorp, The Netherlands; 3 Department of Pediatric Hematology, Immunology, Rheumatology and Infectious Diseases, Emma Children’s Hospital AMC, University of Amsterdam, Amsterdam, The Netherlands; 4 Department of Pediatrics, Hagaziekenhuis Juliana Children’s Hospital, The Hague, The Netherlands; 5 Department of Pediatric Rheumatology, Amsterdam Rheumatology and Immunology Center location Reade Amsterdam, Amsterdam, The Netherlands; 6 Department of Pediatrics/Pediatric Rheumatology, Erasmus MC Sophia Children’s Hospital, Rotterdam, The Netherlands; 7 Department of Rheumatology, Leiden University Medical Center, Leiden, The Netherlands

**Keywords:** Juvenile idiopathic arthritis, Treat to target, Window of opportunity, Treatment strategy study, Biologicals, Inactive disease

## Abstract

**Background:**

Combination therapy with prednisone or etanercept may induce earlier and/or more improvement in disease activity in Disease Modifying Anti Rheumatic Drug (DMARD) naïve non-systemic Juvenile Idiopathic Arthritis (JIA) patients. Here we present three months clinical outcome of initial treatments of the BeSt-for-Kids study.

**Methods:**

Included patients were randomized to either: 1. initial DMARD-monotherapy (sulfasalazine (SSZ) or methotrexate (MTX)), 2. Initial MTX / prednisolone-bridging, 3. Initial combination MTX/etanercept. Percentage inactive disease, adjusted (a) ACR Pedi30, 50 and 70 and JADAS after 6 and 12 weeks of treatment (intention to treat analysis) and side effects are reported.

**Results:**

94 patients (67% girls, 32 (arm 1), 32 (arm 2) and 30 (arm 3) with median (InterQuartileRange) age of 9.1 (4.7-12.9) years were included. 38% were ANA positive, 10 had oligo-articular disease, 68 polyarticular JIA and 16 psoriatic arthritis. Baseline median (IQR) ACRpedi-scores: VAS physician 49 (40-58) mm, VAS patient 54 (37-70) mm, ESR 6.5 (2-14.8)mm/hr, active joint count 8 (5-12), limited joint count 3 (1-5), CHAQ score 0.88 (0.63-1.5). In arm 1, 17 started with MTX, 15 with SSZ.

After 3 months, aACR Pedi 50 was reached by 10/32 (31%), 12/32(38%) and 16/30 (53%) (p = 0.19) and aACR Pedi 70 was reached by 8/32 (25%), 6/32(19%) and 14/30(47%) in arms 1-3 (p = 0.04). Toxicity was similar. Few serious adverse events were reported.

**Conclusion:**

After 3 months of treatment in a randomized trial, patients with recent-onset JIA achieved significantly more clinical improvement (aACRPedi70) on initial combination therapy with MTX / etanercept than on initial MTX or SSZ monotherapy.

**Trial registration:**

NTR1574. Registered 3 December 2008.

## Introduction

Juvenile Idiopathic Arthritis (JIA) is the most common auto-immune disease in children [1] except for systemic JIA which is nowadays viewed as an auto-inflammatory disease [2]. Many children suffer from chronic functional disability and damage due to prolonged inflammation [3]. The ILAR criteria divide the heterogeneous disease in 7 categories [4]. Prognosis is difficult to predict and even oligoarticular disease can have a debilitating course [5]. Nowadays an expanding repertoire of disease modifying antirheumatic drugs (DMARD) including biologicals is available for treatment [6]. Evidence-based information is available on the efficacy of individual products [7–15] but knowledge on therapeutic strategies in children is still scarce [16, 17]. As shown in the BeSt study in rheumatoid arthritis patients[18], it is likely and was illustrated previously that also in JIA a window-of-opportunity exists where the disease is most responsive to treatment and susceptible for permanent suppression [11, 16, 17, 19]. Additionally we know that an early response to therapy is related to a better outcome [20, 21].

In the current study we investigate which of 3 treatment strategies is most effective, fast-acting and safe in a randomized clinical trial comparing three initial therapies: arm 1 initial monotherapy with MTX or SSZ; arm 2 initial combination therapy with MTX and prednisolone and arm 3 initial combination therapy with etancercept and MTX. We hypothesized that compared to initial monotherapy (arm 1) with sulphasalazine or methotrexate or initial combination therapy with MTX/prednisone (arm 2) early treatment with etanercept and methotrexate (arm 3) would lead to significantly more and earlier clinical inactive disease.

## Methods

### Patients

Patients diagnosed as DMARD-naive JIA, either rheumatoid factor negative polyarticular, oligoarticular JIA, or juvenile psoriatic arthritis, in need of systemic DMARD therapy according to treating physician, with less than 18 months of complaints, aged between 2-16, were eligible at 5 participating sites in the Netherlands. Patients suffering from rheumatoid factor-positive JIA are preferably treated with combination therapy from the start and were excluded [17] as well as systemic JIA and Enthesitis Related JIA since they comprise of JIA patients with different clinical features potentially increasing heterogeneity. Patients with JIA related uveitis were excluded due to possible exposure to etanercept which is known to be less effective in uveitis treatment [22–26].

### Study design

Data are collected through the BeSt for Kids study, an investigator-initiated multicentre randomised single blinded clinical trial which will have 2 years follow-up in three treatment arms in a treat-to-target setting. The study was approved by the Medical Ethical Committee of the Leiden University Medical Center and local Ethical Committees prior to start at each study site. Written Informed consent was obtained from patients above 12 years of age and parents of all participating patients. Patients were enrolled and randomly assigned to one of three treatment arms by variable block randomization, stratified per center, as oligo or polyarticular disease.

### Initial treatments

Patients assigned to arm 1 started with Sulphasalazine 50 mg/kg up to 2000 mg/day or MTX10mg/m2/wk orally or subcutaneous (sc)(max 25 mg/wk).

Patients assigned to arm 2 started with MTX 10 mg/m2/wk orally or sc (max 25 mg/wk) in combination with prednisolone orally 0,5 mg/kg for four weeks, tapering by 1 week 0,25 mg/kg and 1 week 0,125 mg/kg, then stop.

Patients assigned to arm 3 started with a combination of etanercept 0,8 mg/kg/wk sc and MTX 10 mg/m2/wk orally or sc (max25mg).

Prior to etanercept treatment, all children were screened for tuberculosis by a purified protein derivative skin test and a chest radiograph. All tested negative. Concomitant treatment with non-steroidal anti-inflammatory drugs (NSAIDs) and intra-articular glucocorticoid injections were permitted without a maximum and registered per strategy. Other parenteral glucocorticoids were not allowed. The use of DMARD or oral glucocorticoids was only permitted as dictated by the treatment protocol. All protocol deviations were recorded. All patients received folic acid during MTX treatment.

### Assessment of disease activity: definition of improvement and inactive disease

The core set criteria [27] were scored at 6 weeks and 3 months by a research nurse, physical therapist or pediatric rheumatologist who remained blinded to the allocated treatment group during study period. Since the protocol was written in 2008 inactive disease on medication was defined based on the modified Wallace 2004 definition [28] instead of the current definition [29]. Based on previous results [30] we stated that a doctor’s overall assessment score below 10 mm (instead of 0 mm) on the VAS indicated no disease activity provided that all other parameters as defined [28] indicated inactive disease. We defined ESR values under 16 mm/h as normal.

Definition of improvement was based on ACRPedi30/50/70% [27]. Changes in outcomes that remained within normal limits (ESR ≤ 16 mm/h and VAS physician < 10 mm) were not taken into account in ACRPedi calculations and were corrected for, resulting in adjusted (aACRPedi30/50/70%) scores.

Juvenile Arthritis Disease Activity Score (JADAS)-10 score were calculated as described previously [31]. Delta JADAS10 was defined as the difference between JADAS10 score at subsequent visits with baseline score.

### Toxicity

At each visit (baseline, 6 weeks, 12 weeks), laboratory tests were performed as clinically indicated: complete blood count, liver and kidney function. The treating physician recorded all adverse events (AEs), serious adverse events (SAEs), and if necessary, made treatment adjustments in accordance to the protocol. SAEs were defined as any adverse reaction resulting in any of the following outcomes: a life threatening condition or death, a significant or permanent disability, a malignancy, and (prolonged) hospitalization.

### Sample size calculations

Expected percentages of time to inactive disease were extrapolated from available literature in 2008 [9, 11, 13] and based on estimation. For the comparison of arm 1 versus arm 3, with power > 90% a difference of 10% in arm 1 versus 60% in arm 3 can be detected with two groups of 30 patients assuming a hazard ratio of 8.70, a drop-out rate 20%, a percentage that switched groups 20%, an alpha 0.05, by two-sided log rank test. Based on analogous calculations (PASS2008) two groups of 45 and 54 patients were needed to detect differences between arm 2 versus arm 3 and between arm 1 versus arm 2. Initially 60 patients per arm was aimed for. Due to slow inclusion rate, the study protocol was amended in 2012 to include 3 groups of 30 patients, leaving enough power to compare arm 1 versus arm 3.

### Statistical methods

Missing data in core set variables were scarce (<1%). All available data were included for intention-to-treat analysis. Last observation carried forward was used to deal with few missing values (n = 5). Student’s t-test was used to compare continuous normally distributed variables between groups. Non-parametric Mann Whitney U tests were used otherwise. For dichotomous variables, Pearson’s chi-square test was used. A two-tailed probability value of P < 0.05 was considered statistically significant. P-values were not adjusted for multiple statistical tests.

The Trial was registered in the Dutch Trial Register number 1574.

## Results

### Baseline characteristics

Baseline demographics and disease characteristics of the three groups showed no statistically significant differences and are summarized in Table 1.

**Table 1:** Baseline demographics and disease characteristics

	Arm 1 MTX or SSZ monotherapy (*n* = 32)	Arm 2 Combo MTX+ 6 wks prednisone (*n* = 32)	Arm 3 Combo MTX+ etanercept (*n* = 30)
Age (years)	8.8 (4.8-12.7)	10.2 (6.6-13.9)	8.6 (4.2-12.4)
Symptom duration* (month)	7.8 (5.3-11.6)	5.9 (4.4-13.3)	8.5 (5.0-13.1)
ANA positive (%)	15 (47)	11 (34)	9 (30)
Female (%)	24 (75)	19 (59)	20 (67)
JIA category:			
Oligo (persistent) Poly articular Psoriatic (poly)	5 (3) 22 5	3 (1) 22 7	2 (2) 24 4
VAS physician (mm)	48 (40-55)	50 (39-58)	51 (37-61)
VAS patient/parent (mm)	48 (31-58)	59 (35-74)	58 (39-71)
CHAQ (0-3)	0.88 (0.28-1.50)	0.94 (0.63-1.69)	0.88 (0.75-1.53)
No. active joints	7.5 (5.0-12.5)	7.5 (6.0-11.8)	8.5 (5.8-13.0)
No. limited joints	2 (0-4.5)	2 (1.0-3.8)	3 (1.8-5.0)
ESR (mm/hour)	6.5 (2-11)	6.0 (2-24)	9.0 (4-25)
JADAS-10 (0-40)	15.7 (13.5-20.2)	17.9 (15.2-21.9)	19.1 (13.8-23.2)

All results in medians (InterQuartile Range) unless stated otherwise;*time from first presenting symptoms to inclusion in the study

### Outcome

**Fig. 1:**
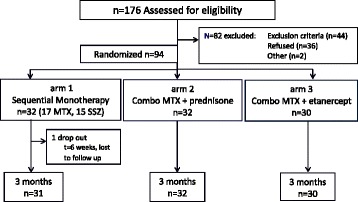
Study profile of the BeSt for Kids study

Figure 1 shows the flow diagram of the study. 94 patients with early JIA, with a median duration between diagnosis and inclusion of 6 weeks (IQR 3-14) and a median duration of symptoms of 7.5 months (IQR 5-12,5), were randomized to one of three treatment groups: 32 patients assigned to monotherapy (arm 1), 32 patients assigned to combination with methotrexate and prednisone-bridging (arm2) and 30 patients were assigned to combination of etanercept and methotrexate (arm 3).

### Adjusted ACRPedi30/50/70 and early inactive disease

Results are summarized in Table 2. From the patients in inactive disease according to our definition: 11/21(52%) had a VAS physician of 0 mm, while 10/21(48%) had a VAS that was scored >0 mm, the average was 3.8 mm.

**Table 2:** Outcome after 6 weeks and 3 months in BeSt for Kids study

	Arm 1 Sequential monotherapy *n* = 32	Arm 2 Combo MTX + 6 wks prednisone *n* = 32	Arm 3 Combo MTX+ etanercept *n* = 30	*p*
Inactive disease (%)* 6wks 3 mths	0 (0) 8 (25)	4 (13) 3 (9)	1 (3) 5 (17)	0.25
aACR Pedi 30 (%) 6 wks 3 mths	15 (47) 16 (50)	18 (56) 17 (53)	17 (57) 22 (73)	0.68 0.13
aACR Pedi 50 (%) 6wks 3 mths	9 (28) 10 (31)	14 (44) 12 (38)	11 (37) 16 (53)	0.56 0.19
aACR Pedi 70 (%) 6wks 3 mths	3 (9) 8 (25)	8(25) 6 (19)	6(20) 14 (47)	0.25 0.04
JADAS-10 (median) 6wks 3 mths Δ JADAS-10 (median) 6wks 3 mths	13.9 9.0 3.2 6.9	9.6 11.5 6.6 5.7	12.4 8.2 5.0 10.2	0.12 0.25 0.012 0.22

*according to our definition of inactive disease modified to Wallace 2004 definition: no active synovitis, no fever, rash, serositis, splenomegaly or generalized lymphadenopathy attributable to JIA. No active uveitis, ESR ≤ 16 mm/h and physician’s VAS <10 mm

### Medication changes and protocol violations

Medication changes and protocol violations are summarized in Table 3. In arm 1 and arm 2 more medication changes occurred compared to arm 3 in the first three months of therapy due to adverse events (*n* = 5). Use of prednisone outside of protocol occurred 3 times in arm 1. Of the 15 patients who started on SSZ, 3 switched to MTX after 6 weeks due to nausea, malaise, headache.

**Table 3:** Medication changes and protocol violations in first 3 months

	Arm 1 MTX or SSZ monotherapy *n* = 32	Arm 2 Combo MTX+ 6 wks prednisone *n* = 32	Arm 3 Combo MTX+ etanercept *n* = 30
MTX dose reduction/switch to SC	2	1	2
Switch SSZ to MTX	3/15	NA	NA
Corticosteroids outside of protocol -kenacort intramuscular -prednisone orally 4-6wk	3 2 1	0 0 0	0 0 0
Intra articular corticosteroid injections	0	0	0

*SSZ* sulphasalazine, *MTX* methotrexate, *sc* subcutaneous, *IM* intramuscular, *NA* not applicable

### Adverse events

A summary of toxicity is given in Table 4. A total of 28% (26/94) of all patients experienced ≥ one AEs: 7/32(22%), 9/32 (28%) and 10/30(33%). Gastro-intestinal symptoms were most frequently reported and were observed 7/32 (22%), 14/32 (44%) and 9/30(28%) in arm 1, 2 and 3. Second mostly reported were mild infectious complications (8/32 (25%)in arm 1, 6/32 (19%) in arm 2 and 13/30 (43%) in arm 3) with 8 upper respiratory tract infections documented in arm 3. Hospital admissions accounted for 3 SAEs in the first three months. One SAE due to viral pneumonia with mild oxygen demand ( on SSZ, arm 1), one patient (on MTX, arm 1) suffered from prolonged vomiting which resolved after admission and stopping of MTX. One patient (on MTX, arm 2) had fever of unknown origin while on MTX and was observed shortly without additional therapy.

**Table 4:** Toxicity in the three treatment arms

Treatment arm	Arm 1 Sequential monotherapy MTX or SSZ *n* = 32	Arm 2 Combo MTX + 6 wks prednisone *n* = 32	Arm 3 Combo MTX + etanercept *n* = 30
Total number of AEs	33	46	39
Number of SAEs	2	1	0
Cardiovascular	0	0	1
Pulmonary	1	2	0
Gastrointestinal	7	14	9
-Nausea	3	8	6
-Vomiting	0	3	1
-Diarrhoea	0	1	1
-Rectal Blood loss	1	0	0
-Liver enzyme abnormality	3	2	2
Neurologic	4	3	2
-Headache	2	0	1
-Sleeping disturbances	1	2	0
-Behavioral problems	1	1	1
Leukopenia	5	1	1
Skin/mucosal membranes	3	4	3
Infectious	8	6	13
-Upper respiratory tract infection	1	1	8
-Gastro-enteritis	0	1	1
-Skin/mucosal infection	1	1	2
-Fever	2	1	1
-Infectious other	1	2	1
General malaise/fatigue	3	5	1
Other	0	3	2

## Discussion

In the BeSt for Kids study, early clinical improvement in patients with early JIA was the aim of the three initial therapies: initial monotherapy with MTX or SSZ, MTX with initial bridging with prednisone, and MTX with etanercept. We found comparable outcomes in all three arms, with the exception that initial combination therapy with etanercept /MTX resulted in a significantly higher percentage of children that had reached aACRPedi70 after three months of treatment. All three groups already after 6 weeks showed improvement, and there was a trend for further improvement in arms 1 and 3, possibly related to discontinuation of bridging therapy with prednisone in arm 2. The effect of prednisone bridging is visible in high aACRPedi 30/50/70% responses after six weeks but improvements diminished after tapering and stopping of prednisone.

Medication changes had occurred more often in arm 1 and arm 2 as compared to arm 3. Toxicity was comparable and acceptable. A subgroup of arm 1 patients performed better than expected by reaching inactive disease after only three months of monotherapy: 4 of them on SSZ and 4 on MTX (25% of all patients in arm 1). Inactive disease after 3 months was rare in arm 2 (9%), and occurred in 17% of patients in arm 3. Outside-of-protocol use of corticosteroids in arm 1 occurred three times in the first three months, these patients did not reach an ACRPedi50 or inactive disease after three months. Apparently for today’s physicians it was hard to hold on to the protocol dictating no additional use of steroids in the current era of impatient doctors and demanding patients, but in this study it helped little to achieve inactive disease.

To minimize the risk of bias of the open design, all outcome measurements were assessed by trained research nurses/physiotherapists/physicians who were blinded to the allocated treatment strategy during entire study period.

Limitations of our study are the relatively small sample size because of slow inclusion rate. These results are promising, but follow up is too short to advocate as yet a primary start with etanercept in DMARD naive new onset JIA patients. The BeSt for Kids study will continue with a treat-to-target design, with medication adjustments aiming to achieve and maintain inactive disease, including after tapering strategies in all three arms. Prospective data on follow-up to 24 months in the BeSt for Kids study will include assessment of possible radiographic joint damage and level of physical functioning.

In conclusion, during the first 3 months of the BeSt for Kids study patients with newly diagnosed JIA who received initial combination therapy with methotrexate and etanercept had significantly more aACRpedi70% responses, comparable side effects and fewer medication changes as compared to methotrexate or sulfasalazine alone or methotrexate and 6 weeks prednisone bridging therapy. Long term follow up data on the extension of initial treatments aiming at inactive disease by a treat to target regime, are needed to relate to these initial positive results.

## Abbreviations

AE: Adverse event
Combo: combination therapy
DMARD: Disease modifying antirheumatic drug
ERA: Enthesitis Related Arthritis
IM: intramuscular
IQR: interquartile Range
JIA: Juvenile idiopathic arthritis
MTX: methotrexate
NSAID: non-steroidal anti-inflammatory drugs
SAE: Serious Adverse event
SC: subcutaneous
SO-JIA: Systemic Onset JIA
SSZ: Sulphasalazine
